# Experiences with smoking habits and the need for cessation among habitual smokers in Japan: a qualitative study based on semi-structured interviews

**DOI:** 10.1186/s12875-023-02254-8

**Published:** 2024-01-02

**Authors:** Kae Shiratani, Junko Shimasawa, Mayumi Mizutani

**Affiliations:** 1 Department of Community Health and Public Health Nursing, School of Nursing, The Jikei University, 8-3-1 Kokuryocho Chofu, Tokyo, 182-8570 Japan; 2 Department of Public Health Nursing, Mie University Graduate School of Medicine, 2-174, Edobashi, Tsu, Mie, Japan

**Keywords:** Relapse prevention, Smoking cessation, Smoking habit, Smoking motives, Tobacco dependence

## Abstract

**Background:**

Although more than half of the habitual smokers recognize that they want to quit smoking cigarettes, approximately half have failed to quit and experienced distress relapse; therefore, there is an urgent need to focus on these populations. When chronic behavior occurs, it is necessary to view the behavior in the context of the entire life of the person involved, considering the history of the person. In this study, we aimed to describe experiences with smoking from the onset to the present and the need for smoking cessation among habitual smokers in Japan and to explore efforts to address them.

**Methods:**

Semi-structured interviews that lasted for 55–90 min were conducted with the cooperation of 16 habitual smokers who smoked cigarettes daily. The content of the interviews included demographic characteristics, experiences with smoking from the onset to the present, whether they have attempted to quit and related experiences, and their thoughts on smoking. Interviews were transcribed verbatim and analyzed qualitatively. The Medical Research Ethics Review Committee of Jikei University approved this study (approval number: 33–384(11008)).

**Results:**

The participants were aged 26–59 years (mean ± SD: 40.8 ± 8.9 years) and included 10 men and 6 women. The participants started smoking between age 13 and 24 years. The highest number of cigarettes smoked in the participants’ lives ranged from 10 to 80 daily, and 12 participants had attempted to quit smoking so far without success. Regarding experiences with smoking from the onset to the present, four themes of “expand one’s world,” “unconscious attachment,” “attempts and failures,” and “losing oneself” were extracted. Regarding the need for smoking cessation, four themes of “empowerment from experts,” “peer interaction,” “social commitment,” and “recovery of confidence” were extracted.

**Conclusion:**

To support smoking cessation from the perspective of habitual smokers, in addition to improvements through the existing approaches, it is important to recover their confidence using ongoing activities in peer groups according to the target background and support from experts incorporating visual assessments of lung function, along with multiple short-term goals. It is also necessary to raise awareness in communities through activities.

## Background

Smoking is a major risk factor for disability-adjusted life years (DALYs) [1], cancer [2], cardiovascular diseases [3], respiratory and chronic obstructive pulmonary diseases [4], mental health [5], tuberculosis [6], coronavirus disease 2019 (COVID-19) [7], and other diseases. Despite this, there were 1.14 billion smokers worldwide in 2019, with 769 million deaths and 200 million DALYs wasted owing to smoking [8]. The estimated smoking rate has decreased from 32.7% in 2000 to 22.3% in 2020, mainly because of several key measures implemented by the World Health Organization Framework Convention on Tobacco Control (WHO-FCTC), namely, taxation, smoke-free policies, warning labels, bans on advertising, and cession programs [9]. However, the smoking population continues to increase in some countries [10]. Japan is among the top 10 countries with the highest number of smokers. These 10 countries account for approximately two-thirds of the global smoking population; therefore, smoking cessation is an urgent and important task [8].

Effective methods of smoking cessation include pharmacological therapy [11], nicotine replacement therapy [12], and various psychological therapies such as counseling [13, 14] and cognitive behavioral therapy [15, 16]. Combining these pharmacological or nicotine replacement therapies with psychological therapy has been found to be the most effective [17, 18], and many habitual smokers are attempting to quit on their own, one way or another. Particularly, a systematic literature review on changes in smoking behavior associated with COVID-19 confirmed large purchases and consumption of cigarettes owing to the lockdown, while smoking abstinence attempts and motivation have also increased [19], indicating a growing need for smokers to abstain. More than 70% of smokers in Japan intend to quit or reduce their smoking in 2019 [20]; however, another nationwide survey of habitual smokers in 2016 found that the percentage of smokers who attempted to quit during a year was 30%, and of these, half failed to quit smoking [21]. Similarly, there are numerous reports of long-term smoking cessation rates of approximately 50% worldwide [22, 23], depending on the population of trial smokers. The success rate of smoking cessation varies widely by region and social life, and it is essential to make a comprehensive effort for both individual smokers and the entire group. Efforts to enable smokers with diverse backgrounds to abstain from smoking temporarily and continue to abstain from smoking for a long period to prevent relapse are urgent.

In Japan, smoking control measures have been a top priority, according to the WHO-FCTC, and have also been emphasized in the Sustainable Development Goals. However, the laws and regulations corresponding to this convention are not yet in place. Furthermore, Japan is one of the countries worldwide lagging in taking countermeasures against smoking [24] because pressure from the tobacco industry, consideration for smokers, and tax revenues make it difficult to promote a smoke-free policy [25]. Throughout Japan, the Health Promotion Law, a related law, has been revised, but only to strengthen the prevention of passive smoking in some indoor areas [26]. In Tokyo, an advanced ordinance was enacted in time for the 2020 Olympic and Paralympic Games (held in 2021), banning smoking indoors in principle [27]. Japan’s average smoking rate of 16.7% in 2021 remains high among developed countries and is equal to the average smoking rate of The Organization for Economic Cooperation and Development countries in the same year [28]. Furthermore, the smoking rate among Japanese males in their 40s exceeded 36.5% in 2019, and 26.7% of smokers use heated cigarettes regularly, particularly among young and middle-aged people in their 20s–50s in Japan [20]. The risks of heated cigarettes are underestimated among smokers, and issues related to smoking withdrawal are becoming more obscure and complex. Therefore, appropriate methodologies should be considered, especially in studies that focused on behavior and attempt to teach skills to cope with smoking cravings [29].

Smoking is a tobacco addiction and a chronic behavior. When chronic symptoms and behavior occur, it is necessary to consider the behavior in the context of the entire life of the parties involved in the process [30]. In recent qualitative studies, individuals who smoked were asked directly about their smoking behavior, socio-cultural and environmental factors influencing smoking [31], perspectives on smoking habits [32], factors affecting smoking initiation and cessation [33], reasons for failure to quit smoking [34], and experiences of attempts to quit smoking [35]. However, the results of each study were based on the cultural background and living environment of the country or region and are expected to differ between countries or regions. Therefore, to examine smoking cessation support for habitual smokers in Japan, it is necessary to explore the smoking experiences and cessation need of this population.

In this study, we aimed to describe experiences with smoking from the onset to the present and the need for smoking cessation among habitual smokers and to discuss smoking cessation support from the perspective of the individuals involved. The consideration of the care process for continued abstinence from smoking, which is frequently accompanied by relapse, can contribute to the development of primary care science from the perspective of building partnerships with those who treat chronic symptoms and the inclusiveness of the care of those involved. It can also provide a methodology for recovery from chronic behavior and symptoms.

## Methods

### Design

This was a qualitative descriptive study based on semi-structured interviews with current habitual smokers.

### Participants

Participants were recruited using public offerings by distributing research cooperation flyers, which indicated “It is not necessary to be interested in quitting smoking, tell us about your smoking experience, we respect your experience,” at a community center and business establishments and through snowball sampling methods between March and October 2022. The inclusion criteria were (1) those who consented to the study and cooperated in the interview, (2) current habitual smokers who smoked multiple times daily, and (3) those aged between 20 and 60 years, considering the Law on the Prohibition of Smoking by Persons Under 20 Years of Age and a sharp decline in smoking rate after the age of 60 years, following retirement in Japan.

### Data collection

The study was explained to the potential participants who agreed to participate in the study, and their consent was obtained on paper; interviews were conducted using Zoom Video Communications with eye contact on the screen. Interviews lasted between 55 and 90 min. According to Strauss et al. in 1984, a comprehensive understanding of individuals with chronic symptoms and behaviors involves considering not only their physical symptoms but also the trajectory of behavior, psychological aspects such as emotions and values, social aspects such as interactions with family and affiliations, and the person’s needs [30]. Therefore, we proposed two research questions: “What are the experiences of habitual smokers from the onset to the present?” and “What do habitual smokers need to continue abstaining from smoking based on these experiences?” Information on demographic characteristics, such as gender, age, education level, current cohabitants, cohabitant parent(s) until adulthood, cohabitant smoker(s) until adulthood, smoking history, comorbid diseases and symptoms, and other habits, were collected. Smoking experiences included (1) smoking experiences from the onset to the present and (2) thoughts about smoking. Smoking cessation need included (1) smoking cessation attempts, (2) factors that proved helpful and challenges encountered in the attempt to quit smoking (for participants who had not attempted cessation, anticipated factors that might influence their decision to quit, both positively and negatively), and (3) desired initiatives and environmental improvements toward smoking cessation. All interviews were recorded with permission and were transcribed into verbatim data.

### Analysis

Verbatim transcripts of the open-ended interviews were prepared for qualitative analysis. First, the responses to the questions were categorized into chunks of sentences, and codes were created to express them in a straightforward manner from the smallest unit of sentence whose meaning could be understood. Next, the multiple codes were classified according to similarities and differences in meaning. Two researchers performed these tasks. Subsequently, sub-themes were generated from codes, and interpretations between themes and sub-themes arising from the interrelationships between concepts were verified. Data were collected and interpreted until no new sub-themes emerged after considering new interviews, which implied that no more semantic content leading to new sub-themes could be obtained. One additional interview was conducted from the state of no emergence, and the interview was terminated when no new meaning was obtained to guide new sub-themes. We also emphasized in vivo codes/concepts, which were extracted using the expression of the narrative content to take advantage of specific expressions in the data. Finally, in the theme generation phase, we repeated our efforts to condense the meanings contained in the themes to create real expressions for all participants. To ensure the validity of the analysis, member checking was conducted. Subsequently, we attempted to refine the themes through repeated modification.

### Ethical considerations

This study was approved by the ethics committee of Jikei University [approved January 2022; no. 33–384(11008)]. Before conducting the interviews, research and ethical considerations were explained to all participants, and written informed consent was obtained.

## Results

### Demographics and smoking-related characteristics of participants (tables 1 and 2)

**Table 1:** Demographics and smoking-related characteristics of participants (N = 16)

Items		Mean ± SD or Numbers
Age		40.8 ± 8.9
Gender		
	Male	10
	Female	6
Latest education		
	High school	4
	College	5
	University	7
Current cohabitant		
	Spouse and others	6
	Spouse	4
	Children or parent(s) without spouse	3
	Alone	3
Cohabitant parent(s) until adulthood		
	Both parents	12
	Single parent	4
Cohabitant smoker(s) until adulthood		
	Yes	14
	No	2
Current passive smoking		
	Workplace	3
	Residence	2
Starting age of smoking		
	Mean age	17.6 ± 3.0
	<20 years	11
	≥20 years	5
Highest number of cigarettes smoked per day in one’s lifetime		
	Mean number	26.4 ± 18.7
	<20 cigarettes	4
	≥20 cigarettes	12
Current number of cigarettes smoked per day		
	Mean number	14.6 ± 6.2
	<20 cigarettes	9
	≥20 cigarettes	7
Current type of smoking		
	Cigarette	8
	Heated tobacco	4
	Mixed of cigarette and heated tobacco	4
Number of quit attempts		
	Never	4
	Once	4
	More than twice	8

SD, standard deviation

**Table 2:** Participant characteristics (N = 16)

Gender	Age group	Current cohabitant	Cohabitant parent(s) until adulthood	Cohabitant smoker(s) until adulthood	Start age of smoking	Highest number of cigarettes smoked per day in one’s lifetime	Current number of cigarettes smoked per day	Number of quit attempts	Comorbid diseases and symptoms	Other habits
F	30s	Two children	Father, mother		13	20	20	1	Rhinitis	Coffee >1000 ml/day, alcohol >70 g/day
M	30s	Spouse	Mother	Mother	15	80	20	>10	Lumbago	Coffee >1000 ml/day, alcohol >100 g/a time
M	40s	Spouse, three children	Mother	Mother, brother	18	20	20	Never	Gastritis	
F	50s	Spouse	Mother		19	20	10	3	Hypertension, high cholesterol	Alcohol >20 g/day
M	30s	Spouse, child	Father, mother	Father, mother	21	20	15	3		
F	50s	Spouse, child	Father, mother	Father, sister	24	30	15	3	Headache, rhinitis, arthritis	
M	20s	(Living alone)	Father, mother	Father, mother	20	10	5	1	Headache, tonsillitis	
M	30s	Spouse	Mother	Grandfather	20	10	10	2		
F	50s	(Living alone)	Father, mother	Father, brother, sister	17	20	8	Never	Hypertension	Alcohol >100 g/a time
M	40s	Mother	Father, mother	Father	15	30	10	Never	Hypertension, high blood sugar	
M	40s	Spouse, child	Father, mother	Father	13	60	25	2	Depression	Alcohol >60 g/day
M	40s	Spouse, three children	Father, mother	Father, mother, sister	17	30	20	1	Sleep disorder	Sugar / Cal. 0 coke >4000 ml/day
F	40s	Spouse, child	Father, mother	Father	18	10	10	5	Hypertension	
F	30s	Father, mother	Father, mother	Father	20	12	6	1		Alcohol >60 g/a time
M	30s	(Living alone)	Father, mother	Father, mother	17	25	20	2		Coffee >500 ml/day
M	30s	Spouse	Father, mother	(Father*)	15	25	20	Never		Sugar / Cal. 0 coke >500 ml/day

*Smoked when the participant was 5 years old

Participants were aged between 26 and 59 years (mean ± SD: 40.8 ± 8.9 years), and 10 were men; one participant was a part-time worker, and the rest had full-time jobs. For education level, seven were university graduates, five were college graduates, and four were high school graduates. Four participants were raised by a single parent during their childhood. Furthermore, 14 participants resided with families (mother or father or grandfather) who were smokers. The starting ages of smoking were from 13 to 24 years. Eleven participants started smoking before the age of 20 years when smoking was permitted under Japan’s law.

The highest number of cigarettes smoked in a single day in the participants’ lives ranged from 10 to 80 daily, and a few had recently changed from heated tobacco products to cigarettes. Twelve participants had attempted to quit smoking without success. Eleven participants had comorbid diseases, and some may have had other habits, such as alcohol or caffeine.

### Experiences of smoking habits among habitual smokers in Japan (table 3)

**Table 3:** Experiences of smoking habits among habitual smokers in Japan (N = 16)

Themes	Sub-themes	Main narrative content
Expand one’s world	Interest in cigarette	Cigarettes were made famous by movies and F1. Actors made smoking look cool. I admired them and longed to smoke. Once I applied for some prizes, I got a Zippo lighter, which made me feel even cooler.
		I used to get many sample cigarettes of a new product from the convenience store I was working at, and I thought I should try smoking. In retrospect, if I had not smoked then, I would not be smoking now.
	Resistance to passive smoking	As I remember, my parents usually smoked in the living room, and I was constantly exposed to smoke. I hated smoke so much then, but when I eventually tried it, I realized that I could smoke.
		The truck I had my job was always filled with a lot of smoke from my co-worker. I didn’t understand why I had to go through this. I thought to myself if I had to passively smoke, I would still rather smoke actively.
	Belonging to the peer community	I went to a summer camp with my clubmates, and they offered me a cigarette. I was included in the group without resistance through smoking, and spending time with them was satisfying.
		To explain, it is like a small group of smokers hanging out together. The community is more important to us, and the small group size makes it more cohesive. There is a sense of sharing space and time.
	Embodiment of oneself	People told me, “wow, that is such a strong cigarette you smoke,” and I pretended to be cool saying things like, “this is normal for me to smoke this much.” I was acting brave.
		I wanted to smoke without worrying about what others would think. Even if I was a little uncomfortable with my surrounding atmosphere, I smoked with the feeling of, “I won’t lose.”
Unconscious attachment	Perceive special sensations	Probably because when I smoke, my blood vessels constrict. I feel dizzy and lightheaded, which is good. It’s like taking a big, fat breath.
		I felt dizzy in my head and a kick in the throat. My lungs would get a little heavier, which was good. It is comparable to how a beer goes from bitter to good.
	Switching their own mood	I was really busy with my double job, in a hurry to make money, and working hard and alone. I wanted to smoke and take a break after work.
		When I am tired or need to take a break from work, I take a deep breath in with a cigarette and exhale deeply to reset. Thus, in a way, it may be similar to deep breathing.
	Calm the unrest	I wake up and smoke; eat a meal and smoke; and brush my teeth, change clothes, and smoke. I smoke 10 cigarettes before work. That is my rhythm, and it calms me down. I feel better prepared for work.
		I would rather smoke than worry about something; it is a tranquilizer. I cherish this one important time because I don’t know when and where I will be able to smoke again. If I have time, I will surely smoke.
Attempts and failures	Reflect on one’s own smoking habit	I found myself smoking while lounging around, when using my phone, and when I’m thinking. It is no longer a small break or social lubricant I once thought it was. It is no longer a benefit.
		I guess I am not that attached to cigarettes. However, I look at my friends, and the amount they smoke is amazing. Seeing them makes me think I could always quit smoking.
	Explore smoke reduction and cessation	I searched the Internet for a surefire way to quit. I thought that the success rate of using varenicline was relatively high. I read all the success stories of others. Therefore, I decided to use varenicline.
		I attempted to quit smoking cigarettes by putting candy or gum in my mouth to drown my loneliness. However, when I do so, I had to share it with everyone every time. In addition, I gained weight.
	Receiving an invitation to smoke	A senior gave me heated cigarettes and said, “I heard you quit smoking; this is good.” I was so amazed at the quality of the heated cigarettes that I repurchased them that same day.
		At a drinking party, he asked me to smoke, and I thought, “I had quit tough. It is a drinking party; it is OK.” I smoked. Fast forward, I started smoking again with my seniors during breaks.
Lose oneself	Outflow of time and money	I don’t know why I pay to smoke. If I could stop, I would be able to save $5, and use it for buying a meal instead. However, my body craves for smoking and I want to do something habitually. I don’t know.
		Cigarettes have become the center of my life. I come to work early to smoke, go out for easy lunch and smoke, and come back early from lunch to brush my teeth to eliminate the smell of cigarettes.
	Trust wavers	My spouse was very angry when she learned about my smoking relapse. I was treated like a criminal. I could neither talk to anyone about it. In fact, we stopped talking together.
		My father died early because of smoking. Because of that, my mother would say, “you stink,” and shunned me whenever I smoke. She would also call me a smoking “douche.”
	Shrinkage of whereabouts	There used to be a community of smokers where I had the opportunity to connect and talk with older people at work. Now, there is no place to smoke or friends, and I smoke alone.
		It used to be that if you had never met them in the smoking area, they would say, “I have got a new release. Do you want one?” kind of conversation. Very nice place, but it is becoming lesser nowadays.
	Doubt about oneself	I paid for varenicline treatment, but I started smoking again. I betrayed not only the medicines but also myself. I want to win the battle against myself. Next time I will have to fight on my own.
		I thought I could quit, but I could not. I smoked again. I even regretted why I said, “I quit.” No matter how hard I tried, I just cannot stop. It is very painful and hard. I deceived myself, and lost the trust of my wife.

Four themes were identified as experiences with smoking from the onset to the present from the semi-structured interviews conducted among the participants. Table 3 is ordered according to themes, sub-themes, and the main narrative by participants representing the themes and sub-themes. The following describes the four themes and the sub-themes with double quotations, with each theme also explained based on what was stated by the participants.

1. Expand one’s world.

Participants developed an “interest in cigarettes” through the brands made famous by Formula One, movies, those smoked by their favorite celebrities, or through those recommended by their friends or part-time jobs. They also experienced “resistance to passive smoking” indirectly through their parents and siblings who smoked at home and the poor passive smoking environment at their part-time jobs. They also experienced “belonging to the peer community” by accepting invitations to spend time and space with their seniors and club mates. In addition, they experienced the “embodiment of oneself” by smoking cigarette brands with high nicotine and tar content or brands they were particular about. Thus, they expand their relationships and worldviews as “expand one’s world.”

2) Unconscious attachment.

When participants started smoking, they experienced a special sense of weight in their lungs and throat caused by the effects of cigarette inhalation and a momentary loss of consciousness due to vasoconstriction, which caused them to “perceive special sensations.” They had also experienced “switching their own mood” to divert their mind from hectic work and constant child-rearing. Gradually, they transcended to the experience of “calm the unrest,” where they implored themselves to pause for a cigarette each time, they completed a task. Thus, they developed the habit of “unconscious attachment,” where they could not let go of the cigarettes.

3) Attempts and failures.

Participants experienced “reflect on one’s own smoking habit,” where they reflected on the physical effects of their smoking and the meaning of smoking. They attempted to reduce the number of cigarettes they smoked and substituted candies, gum, and carbonated drinks for smoking. They visited a smoking cessation clinic to understand the effects of smoking cessation and to “explore smoke reduction and cessation.” However, they experienced “receiving an invitation to smoke” when they were invited by their friends or seniors to smoke, they started smoking again. Thus, they experienced “attempts and failures” when they repeatedly made an effort to quit smoking and relapsed.

4) Losing oneself.

Participants realized that they were spending more on buying cigarettes, taking breaks to smoke cigarettes, leaving earlier in the morning, having shorter lunches, and other time-consuming “outflow of time and money” experience. They also experienced “trust wavers” when their families disapproved of their smoking relapse, and loved ones were severely disappointed when they found out about their relapse. They also experienced a “shrinkage of whereabouts” as more of their friends succeeded in quitting smoking, and they were forced to smoke in secluded areas with silence and alone. Finally, they experienced “doubt about oneself,” where they refused to talk openly with those around them and became suspicious of their inability to stay away from cigarettes. They had experienced “losing oneself” through the above experiences.

### Need for smoking cessation among habitual smokers in Japan (table 4)

**Table 4:** Need for smoking cessation among habitual smokers in Japan (N = 16)

Themes	Sub-themes	Main narrative content
Empowerment from experts	Attitude of trying to understand	It was nice to be able to talk one-on-one. I talked directly with the psychologist and the public health nurse, and because of that, I was able to keep going to them. They were very accommodating.
		I went to the wrong clinic (not right for me). The mood there was very cold. They looked at me like I had weak willpower, and even told me that they would find out if I smoked (causes of failure).
	Symptoms relief	When I was taking varenicline, I strangely didn’t enjoy smoking. I thought, “I’m going to quit,” and I kept taking the drug for a while. I didn’t smoke for a while.
		Smoking is always on my mind, and I can’t stop thinking about it. It would be helpful if something could distract me or something to ease my mind, such as an exercise or an idea.
	Praise for efforts	The nurse and doctor were very complimentary about my continued abstinence of smoking. They encouraged me to increase the number of days without smoking and not quit smoking at once.
		The nurses and doctors may have been just doing their jobs, but they were very kind and supportive to me. That made me think, “I have to do my best.”
	Visualize accomplishments	My lung capacity was measured every time I went to the clinic. I could see that my lungs were recovering slowly, and seeing the results made me want to continue to abstain from smoking.
		It is more encouraging to be told that my lung function numbers have improved, or to be told how many days I have been able to quit smoking than to say “I quit for three days” on my own.
Peer interaction	Express weaknesses	I know I’m an addict, I know I shouldn’t smoke, I know it’s tough to not keep smoking, I know I want to quit, but I can’t. So I need a comrade I can say so, I can do it with, or no enemies.
		I tried varenicline and everything else. I’m stuck in my current state with the feeling that the next time I have to do it will be on my own, and it’ll be too hard.
	Forward together	I want to continue to abstain from smoking with other colleagues. And short term goals, like let’s all work together, I think it’s easier to succeed than trying in solitude.
		I wish there was some kind of self-help group. Even if we don’t know who they are or where they are from, we can meet them and work together. I think just not smoking while we talk is an even effect.
	Mutual acceptance	It’s normal to make mistakes of smoking again. It does not suddenly come to zero, but just the fact that I smoke less often makes a difference from before. I want you to look at it with an open mind.
		When people are not acknowledged, their self-esteem decreases. It is very reassuring to have someone who is willing to confront me for putting up with or suffering from not smoking.
Social commitment	Public understanding	I feel persecuted by the media and all things they say about passive smoking. They don’t understand the suffering smokers endure. I’ve switched to heated cigarettes, and it has eased the guilt for me.
		I may be being paranoid, but I thought people would think I had bad manners or that I couldn’t control myself. Smoking is seen as a terrible bad thing in the world, and it’s hard to shrug it off.
	Ideal environment	I would love to be transferred to an environment where I can’t legally smoke, or where everyone around me doesn’t smoke. Then, I can’t smoke either and will eventually quit.
		I haven’t been told I have lung cancer yet, so when the doctor tells me I’m going to die, I’ll change. Besides, if they don’t sell me cigarettes, I could quit.
	Political involvement	If the government wants to reduce the number of smokers, their voices are not being heard. The law is also vague. Stores sell a lot of them. I don’t see they want us to stop or not.
		They are taxing us so much, eliminating most of the smoking areas, but leaving a few, that’s the anger, why don’t they just abolish it all together? Why do they treat smokers so badly?
Recovery of confidence	Realize one’s own efficacy	If there is a cigarette in front of me, I know I wouldn’t feel normal, unfortunately. If I’m drunk and invited to have one, I’ll smoke “just one.” In short, I have to keep bounding myself no to smoke.
		I wonder if I can really quit, since I have started smoking again many times before. I’m just going to keep going through the days of not smoking until I die.
	Free from bondage	The guilt I feel when I smoke, the weird feelings when I don’t, the guilt I feel when I start smoking again, not just the cigarettes, all of that would be gone, and I would be free.
		While I wasn’t smoking, the thought of wanting to smoke kept coming, I kept thinking about cigarettes at all hours. I’ll never be able to stop smoking. I want to be free from smoking.
	Compassion for neighbors	The most important thing is not to start smoking. Quitting in the middle is the hardest thing. I don’t want my children to smoke. I think Taspo system (which is a card identifying adults) is a good idea.
		Compared to before, when smokers smoked at their office desks, they now smoke in a limited number of places, opportunities for people to passively smoke have dramatically decreased, a very good thing.

Four themes were extracted as need for smoking cessation from the semi-structured interviews among habitual smokers. Table 4 is ordered according to themes, sub-themes, and the main narrative by participants representing the themes and sub-themes. The following describes the four themes and the sub-themes with double quotations, with each theme also explained based on what was stated by the participants.

1. Empowerment from experts.

“Empowerment from experts” was the need, which included “attitude of trying to understand,” where people who have been physically, mentally, and socially debilitated by smoking for a long time are sympathized with and healed from the difficulties of smoking cessation through consultations and understanding with doctors, nurses, and public health nurses at smoking cessation clinics and health centers. Participants also required “symptom relief” through medication, nicotine replacement therapy, and teaching relaxation techniques to ease nicotine withdrawal symptoms. Furthermore, participants needed “praise for efforts” from experts as they continued to abstain from smoking. They also needed to “visualize accomplishments” by gradually increasing the number of days of smoking cessation to realize that their short-term goals could be achieved gradually and by periodically measuring lung capacity to visually realize the changes in the lungs due to smoking cessation.

2) Peer interaction.

“Peer interaction” was the need which included “express weaknesses” of expressing the anguish of being unable to quit, the failure to quit smoking, and the pain of withdrawal symptoms. In addition, there was a need for “forward together,” a group of people who could meet face to face, even if they did not know each other, and discuss the progress of their multiple individual short-term goals together. They also needed “mutual acceptance” to understand each other’s feelings of guilt, the pain of relapse, and feelings of alienation from the response of those around them.

3) Social commitment.

“Social commitment” was a need with a view to the surrounding communities and country. There was a need for “public understanding” to understand their situation, such as it was not necessarily initiated solely by their intentions, the pain of being unable to quit smoking, withdrawal, the view of others as a source of passive smoking, and the pain of being considered incapable of self-care. There was also a need for a legal ban on smoking and an “ideal environment” where people could not smoke at all, including restrictions on the sale of cigarettes. Finally, there was a need for “political involvement” because of frustration and anger among the smokers that the government has not banned or supported smoking cessation while permitting the selling of cigarettes in stores and reducing the number of smoking areas. They felt that if the government wanted to reduce smoking, no message was being sent to smokers. They complained that they were being criticized as people who paid high taxes and ignored passive smoking.

4) Recovery of confidence.

“Recovery of confidence” was the need which included “realize one’s own efficacy” to increase self-efficacy by abstaining from smoking and building one’s achievements. In addition, there was a need to be “free from bondage,” i.e., to be free from withdrawal symptoms, the guilt of smoking, and the lack of confidence in being a smoker. Moreover, there was the need for “compassion for neighbors,” which meant caring for oneself and one’s family and neighbors.

## Discussion

This study described smokers’ experiences with smoking from the onset to the present and their need to continue abstaining from smoking based on these experiences. Below is a discussion of the characteristics of the study participants and the results.

Regarding study participants, four were raised by single parents during childhood, most of them had a family member who smoked until their adulthood, and more than half of them started smoking in their teens (the legal age to start smoking in Japan is 20 years old). Parental separation and nicotine dependence of parents in childhood are major examples of Adverse Childhood Experiences (ACEs); several studies related to ACEs have reported a correlation between the severity of ACEs and smoking behavior from teenage years [36, 37], particularly among mothers, which was considered high and overlapped with previous studies on ACEs [38]. Unfortunately, 12 participants had attempted but failed to quit smoking, with some making multiple such attempts. Moreover, the results of this study are similar to those of a previous study that found that the younger the age of smoking initiation and the more cigarettes smoked, the more difficult it was to successfully abstain from smoking [39], suggesting the need to strengthen anti-smoke education to ensure that no one is left behind including school-aged and adolescent individuals. Furthermore, in the process of attempting to abstain from smoking, other addictive behaviors, including alcohol, sugar, and caffeine addiction, were also observed, suggesting the difficulty in continuing to independently abstain from smoking. This finding also parallels existing knowledge that smokers are also overlappingly dependent on other substances [40]. The backgrounds of the participants were diverse but consistent with those of previous studies, and it was necessary to consider the contents and venues of the activities based on the participants’ backgrounds.

The experience of the initiation of smoking was triggered not only by the individual’s interest and concern but also by passive smoking when parents smoked at home, exposure to a poor passive smoking environment at a part-time job, and invitations from close friends to join a group. The participants attempted to broaden their worlds, and the initiation of smoking was caused by factors attributable to the environment during their youth. In a qualitative study on smoking initiation among adolescents, the factors of peer pressure, imitation of parents who smoke, and masculine curiosity were reported, and our study reported similar findings [31, 41]. “Unconscious attachment,” “attempts and failures,” and “losing oneself” were repetitive experiences of attempting and failing to abstain from smoking through a process of addict formation where smoking was substituted for something indispensable, an experience that ultimately resulted in the loss of money, time, trust in others, and trust in oneself. Notably, the participants had attempted to abstain from smoking multiple times in the past and experienced the pain of not being free from cigarettes. Therefore, when supporting smokers, it is essential not to isolate them but to engage them thoughtfully in the background of their smoking initiation and the long process that follows.

The need for smoking cessation “empowerment from experts” comprised encouragement to abstain from smoking, praise for attempting and continuing to abstain from smoking, alleviation of withdrawal symptoms through treatment and teaching relaxation methods, and empowerment through visual evaluation of results by quantifying the improvement in lung function and sharing the number of days of continued smoking cessation with multiple short-term goals. Previous studies have also reported the positive effects of psychological support and withdrawal symptom relief through relaxation programs, such as massage and exercise [13], and the endeavor for acceptance and encouragement without criticism [41]. Reports also state that visualizing one’s lung condition and its effects by presenting lung age are effective [42]. Therefore, it is important to provide support and empowerment through multiple means to support those abstaining from smoking. Concerning “peer interaction,” the participants needed a place where they could express their anxieties and frustrations about continuing to abstain from smoking, exchange information, and express their feelings of shame when they repeatedly failed to quit smoking, as well as a place where they could share their experiences with others who have a similar process. This need is similar to the “belonging to the peer community” aspect extracted from the participants’ experience, and it can be inferred that the participants were seeking companionship. Group programs inspired by group dynamics [43] have received attention in previous studies [44] and have been effective, particularly in Asian countries [45]. Therefore, peers may support each other in abstaining from smoking.

The need for neighbors, national, and political commitment was identified in “social commitment.” As shown in the experience of smoking, smokers frequently have considerable suffering in their backgrounds, including the pain of not being able to quit, and this should be understood by the people around them and by society. Previous studies that described the experience of smokers have similarly reported the need for understanding rather than criticism [41]. Japan’s smoking control measures emphasize the prevention of passive smoking [25], while providing inadequate support for smokers. The government should address the health and ethical issues (e.g., tobacco poisoning – “Can we offer poison to people?”) by separating them from concerns about the tobacco industry and tax revenue. Health professionals should also focus on supporting smokers who wish to quit, and adolescent anti-smoking measures should be adopted. The final and overarching need is “recovery of confidence,” which should be addressed concurrently in the same manner as “empowerment from experts,” “peer interactions,” and “social commitment.” In the experience of smoking, the others’ trust in the participant was shaken, resulting in a reduced sense of belonging and, thus, their confidence should be recovered. Smokers suffer more than two or three-fold from their inability to abstain from smoking, and it is essential to promote practices based on research to understand the feelings of smokers and their application of such research. The novelty of this study is that it describes the longitudinal experience of habitual smokers in Japan from the start of smoking to the present. To change a chronic behavior, it is necessary to consider the behavior in the context of the person’s life [30]. Thus, it is significant that we were able to interview and describe the need for smoking cessation based on smoking experiences. These points in the present study differ from those in existing qualitative studies with narratives from smokers [31–35]. Further, this need was identified not only toward professionals but also toward their peers and society. The importance of meeting this need in an integrated manner and regaining self-confidence was also considered.

As suggestions for program development, in addition to the conventional approaches of symptom relief and psychological support [13], it is important to incorporate a visual assessment of lung function along with the multiple short-term goals and to develop ongoing activities (place and content) in peer groups that are appropriate to the target background, and involve smokers in recovering their confidence. Simultaneously, it is necessary to educate adolescents, society at the community level, and political bodies about the situation of smokers to not only focus on anti-smoking measures.

### Limitations and future studies

Because this study collected data based on the snowball sampling method and the distribution of flyers at a few establishments, it was assumed that the population was somewhat homogeneous regarding employment status, income, and other factors. It is also possible that some of the applicants who saw the flyer were interested in quitting smoking. Furthermore, participant narratives of smoking initiation may have been subject to recall bias. These may have influenced the results. Because smokers are a diverse population, it is necessary to determine the actual situation in diverse populations quantitatively.

## Conclusions

The habitual smokers’ experiences with smoking from the onset to the present include “expanding one’s horizons,” “unconscious attachment,” “attempts and failures,” and “losing oneself.” In contrast, their need for abstinence requires “empowerment from experts,” “peer interactions,” “social commitment,” and “recovery of confidence.” Therefore, to support smoking cessation from the perspective of habitual smokers, in addition to improvements through the existing symptom relief and psychological support approaches, it is important to develop a program that incorporates the visual assessment of lung function, with multiple short-term goals and ongoing peer group activities. This not only helps to continue smoking cessation but also restores the confidence of the smokers. The program should also provide community education that is appropriate for the target audience.

## Data Availability

The datasets generated and/or analyzed in this study are not publicly available to protect the participants’ personal information and to guarantee that confidentiality was maintained during the informed consent process. Anonymized and de-identified data may be queried with the corresponding authors with reasonable request.

## Abbreviations

ACE: adverse childhood experiences
COVID-19: coronavirus disease 2019
DALYs: disability-adjusted life years
WHO-FCTC: World Health Organization Framework Convention on Tobacco Control
